# Report on Three Patients with Blastic Plasmacytoid Dendritic Cell Neoplasm

**DOI:** 10.4274/tjh.2018.0041

**Published:** 2018-08-05

**Authors:** Hale Bülbül, Nazan Özsan, Mine Hekimgil, Güray Saydam, Mahmut Töbü

**Affiliations:** 1 Ege University Faculty of Medicine Hospital, Department of Internal Medicine, Division of Hematology, İzmir, Turkey; 2 Ege University Faculty of Medicine Hospital, Department of Pathology, İzmir, Turkey

**Keywords:** Blastic plazmositoid dentritic cell neoplasm, HyperCVAD regimen, Stem cell transplantation

## To the Editor,

Blastic plasmacytoid dendritic cell neoplasm (BPDCN) is a rare, clinically aggressive tumor that was classified as a distinct entity among myeloid neoplasms in the World Health Organization’s 2016 revision of the classification of acute myeloid leukemia and related neoplasms. Most patients present with cutaneous lesions with or without bone marrow involvement and leukemic dissemination. The tumor cells express CD4, CD56, CD123, and TCL1 [1]. In general, acute lymphocytic leukemia (ALL)/lymphoma-type regimens were reported to show better survival outcomes than acute myeloid leukemia (AML)-type regimens. Complete remissions were registered for 7 of 26 patients after AML-type regimens and 10 of 15 patients after ALL/lymphoma-type regimens, with a significant advantage for the ALL/lymphoma-type approach [2,3]. Patients who were treated with hyper-CVAD showed an objective response, but the duration of response was short so hematopoietic stem cell transplantation (HSCT) should also be considered [4]. Recent interest has been directed towards SL-401, a novel immunotherapy directed at IL-3R, notably overexpressed in BPDCN as well as other myeloid malignancies. This led to the development of SL-401 as an IL-3-diphtheria toxin conjugate that has demonstrated promise for BPDCN in early-phase trials [5,6,7,8]. We aim to share our experience with BPDCN due to its rareness and the lack of a consensus about treatment.

**Figure 1:**
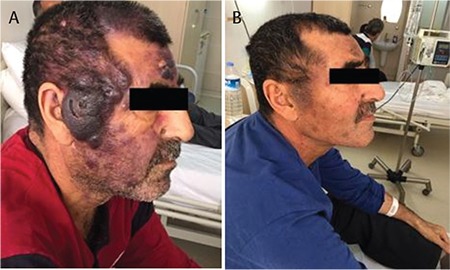
Brown-purple tumoral mass of 3x3 cm in diameter on the right temporal region (A). After a single cycle of chemotherapy, skin lesions regressed (B).

All three patients were male, aged 19, 55, and 65 years, and were admitted to the hospital with fever, weight loss, weakness, and lymphadenopathy. Physical examination revealed that all of them had lymphadenopathies, one of them had hepatosplenomegaly, and two of them had skin lesions. Skin lesions were bruise-like brown to violaceous infiltrated plaques on the back and extremities. One patient had a brown-purple tumoral mass and also brown-purple nodular lesions of the head region (Figure 1). Bone marrow and lymph node biopsies showed diffuse infiltration by medium-sized blasts with irregular nuclear contour, slightly large cytoplasm, high mitotic index, and immunohistochemical expression of CD4+, CD56+, CD123+, and TCL1+. Skin biopsies revealed diffuse infiltration by similar cells. One patient had central nervous system involvement that was pathologically proven by cerebrospinal fluid cytology. In one patient’s bone marrow results, 36% *TCF3* and 35% *TEL* gene deletions were detected by hybridization. A hyper-CVAD regimen was initiated for all patients. After one cycle of chemotherapy, two patients achieved complete remission (CR). One patient who achieved CR and the patient who could not achieve CR died of sepsis. The other patient who achieved CR after one course of chemotherapy was treated with three cycles of the hyper-CVAD regimen as maintenance and afterwards he underwent transplantation with peripheral blood progenitor cells from a related mismatched donor. BuCy was administered for the conditioning regimen before transplantation.

Two patients achieved CR with the hyper-CVAD regimen and one of them who underwent allogenic transplantation is still in CR 18 months after diagnosis. BPDCN can go into durable remission with HSCT regardless of the type of the induction regimen. In particular, auto-HSCT in first CR appears to be a reasonable treatment option and may play an important role in improving the outcomes of BPDCN [9]. On the other hand, high-dose therapy followed by allo-HSCT can provide durable disease control in up to 50% of patients and allo-HSCT should be administered in first CR if possible [10]. Allogeneic stem cell transplantation seems to improve the prognosis, but further studies are needed to confirm the place and the indication of this treatment strategy.
